# Fluid percussion trauma to the placental trophoblast triggers multifaceted injury mechanisms, including mitochondrial stress response, immune dysregulation, cytoskeletal, endothelial, and angiogenic signaling cascades

**DOI:** 10.3389/fcell.2026.1834803

**Published:** 2026-09-09

**Authors:** Michael J. Paidas, Emily M. West, Anna Rosa Speciale, Urja C. Patel, Rajalakshmi Ramamoorthy, Swati Kumar, Rebecca Patrizio, Elise Michelle Belkin, Christina Attia, Aleezeh Shaikh, Ganapathi Kandasamy, Nayab Ahmad, Edna Porter, Anis Ahmad, Arumugam R. Jayakumar

**Affiliations:** 1 Department of Obstetrics, Gynecology and Reproductive Sciences, University of Miami Miller School of Medicine, Miami, FL, United States; 2 Department of Biochemistry and Molecular Biology, University of Miami Miller School of Medicine, Miami, FL, United States; 3 Department of Health Sciences, Obstetrics and Gynecology Branch, University of Florence, Florence, Italy; 4 Department of Molecular and Cellular Pharmacology, University of Miami Miller School of Medicine, Miami, FL, United States; 5 Department of Radiation Oncology, University of Miami, Miller School of Medicine, Miami, FL, United States

**Keywords:** immune and inflammatory response, mitochondria, placenta, trauma, trophoblasts

## Abstract

**Introduction:**

Trauma is the leading cause of non-obstetrical maternal death and is linked to adverse pregnancy outcomes, including placental abruption, preterm birth, and stillbirth. Although placental injury is a known consequence of maternal trauma, understanding its direct effect on trophoblast function remains limited. This study aims to examine the cellular effects of mechanical trauma on human trophoblasts *in vitro*.

**Methods:**

Human trophoblast cells (HTR-8/SVneo) were cultured and exposed to controlled mechanical trauma using a fluid percussion injury (FPI) model, previously validated in traumatic brain injury research. Cell viability was measured 48 h after injury with a fluorometric assay kit. Immunofluorescence and Western blot analyses were carried out to assess the levels of mitochondrial markers (TOM20, COX1, VDAC1) and the antiangiogenic factor sFlt-1.

**Results:**

Trophoblasts subjected to trauma exhibited reduced viability compared with controls. Expression of sFlt-1 and SDHA increased in injured cells, indicating a cellular stress response. Western blot results revealed a twofold decrease in TOM20 levels and a slight reduction in VDAC1 levels, while COX1 levels remained unchanged. Moreover, next-generation sequencing of trophoblasts after trauma showed altered gene expression involving several injury pathways, including mitochondrial stress response, immune dysregulation, cytoskeletal changes, endothelial and angiogenic signaling pathways, calcium signaling, metabolic imbalance, and inflammation. These results suggest that mechanical trauma causes mitochondrial dysfunction and disrupts angiogenic signaling in trophoblasts.

**Discussion:**

Trauma induces significant mitochondrial changes and cellular stress, characterized by reduced viability, decreased TOM20 and VDAC1 levels, and increased sFlt-1 and SDHA levels. These patterns resemble those seen in hypoxia-related placental disorders such as preeclampsia, fetal distress, and placental abruption. Understanding these mechanisms may help clarify how maternal trauma contributes to adverse pregnancy outcomes. Further studies using *in vivo* models are required to explore the clinical relevance of these findings.

## Introduction

Trauma during pregnancy is a major concern and the leading cause of non-obstetrical maternal death. About 7% of all pregnant women experience trauma during pregnancy, with the most common incidents being car accidents, falls, and physical assault (Tweddale, 2006). These traumatic events can cause complications such as placental abruption, preterm birth, and stillbirth (Adams et al., 2022). Placental abruption, which happens when the placenta detaches from the uterine wall early, is the most frequent injury to the gravid uterus in cases of trauma and is the main cause of fetal death when the mother survives (Sadro et al., 2012). Despite the seriousness of these outcomes, there is limited understanding of how trauma specifically affects the mother, placenta, and fetus. The variability in trauma’s intensity, frequency, and timing makes this understanding even more complicated.

Our previous research on traumatic brain injury (TBI) has laid a foundation for exploring trauma-related mechanisms during pregnancy. TBI and trophoblast injury are theoretically similar, involving hypoxia-ischemia, mitochondrial dysfunction, and inflammatory cascades. In our prior research on brain cells, we tested 3, 4, and 5 atm and found that 5 atm caused significant mechanical trauma. These studies have also shown that neurons and astrocytes undergo significant swelling in response to mechanical trauma, impairing cellular function and survival (Jayakumar et al., 2014; Jayakumar et al., 2008). Since the placenta plays a key role in fetal development, examining its response to trauma could help identify early injury biomarkers and improve maternal-fetal outcomes during traumatic pregnancies.

Trophoblasts, the main cellular component of the placenta, regulate nutrient transfer and hormone release during pregnancy. These processes are energy-intensive and depend heavily on mitochondrial function. Therefore, damage to trophoblast mitochondria has been associated with negative pregnancy outcomes such as preeclampsia (Muralimanoharan et al., 2016). However, the impact of trauma on trophoblast function has received limited focus, despite its potential to affect fetal development. To explore this, we adapted a mechanical percussion system previously used to model TBI (Jayakumar et al., 2014; Jayakumar et al., 2018) to study the trauma response of human trophoblast cells *in vitro*.

The objective of this study was to analyze the viability, function, and protein levels of trophoblast cells in response to a controlled mechanical trauma simulation. We hypothesized that human trophoblast cells would exhibit impaired cellular function and viability, as observed in previously studied central nervous system cells.

## Materials and methods

### Study design and setting

#### Trophoblasts culture

Trophoblast cells (HTR-8/SVneo; Cat #CRL-3271) were obtained from the American Type Culture Collection (ATCC, Manassas, VA, USA). The cells were cultured in RPMI-1640 medium (company), supplemented with 10% fetal bovine serum (FBS, ATCC, Manassas, VA, USA) and 5% penicillin-streptomycin (Anti-anti), and maintained at 37 °C in a humidified atmosphere comprising 5% CO2 and 95% air. The media were changed every 48–72 h, and the cells were passaged at 70%–80% confluency using 0.25% trypsin/EDTA.

#### *In Vitro* percussion trauma

Mechanical trauma was applied to cultured trophoblasts using a fluid percussion injury (FPI) device originally described by Sullivan et al. (Jayakumar et al., 2014) and later adapted for cell culture by Shepard et al. (Jayakumar et al., 2008). Culture plates were secured in an injury chamber connected to the fluid percussion device via non-distensible Tygon tubing. A weighted pendulum delivered two pulses of 5 atm, each lasting 25 milliseconds, with a 3-s interval between pulses.

#### Cell viability assay

Cell proliferation and viability were evaluated 48 h after trauma using the Cell Viability Assay (Cat# ab102501, Abcam, Waltham, MA, USA). Fluorometric-blue reagent was added directly to each culture well, incubated at 37 °C for 1 h, and fluorescence intensity was measured with the Qubit fluorometer at excitation 530–570 nm and emission 590–620 nm (Invitrogen, Carlsbad, CA, USA). The fluorescence readings were normalized to protein concentration and expressed as relative fluorescence units (RFU).

#### Immunofluorescence staining

Trophoblast cultures were fixed in 100% methanol for 15 min at −20 °C, then incubated in 3% hydrogen peroxide (H_2_O_2_) in phosphate-buffered saline (PBS) for 3 min. Cells were subsequently blocked with 5% bovine serum albumin (BSA) in PBST for 1 h at room temperature. Primary antibodies against SDHA (1:500; Cat# A13852, ABclonal, Woburn, MA, USA) and sFlt-1 (1:500; Cat# 10136-MM03, Sino Biological, Houston, Texas, USA) were applied overnight at 4 °C. After washing with PBST, cells were incubated with the appropriate HRP-conjugated secondary antibodies for 1 h at room temperature. Coverslips were mounted using Vector Shield Antifade Mounting Medium (Cat# H-1000, Vector Laboratories, Newark CA, USA). Images were captured with a Leica Thunder DMi8 inverted microscope equipped with a K3C color camera. The images were randomly collected in a “blinded” manner. At least 10 fluorescent images were captured and were quantified using the Volocity 6.0 High-Performance Cellular Imaging Software (PerkinElmer, Waltham, MA, USA) as described previously (Paidas et al., 2022a).

#### Western blotting

Trophoblast cultures were lysed in a laboratory-prepared buffer containing 10% protease inhibitor cocktail (Roche Bioscience, Palo Alto, CA, USA), and protein levels were measured using the bicinchoninic acid (BCA) assay. Equal amounts of protein were loaded onto gels for electrophoresis and immunoblotting (Paidas et al., 2022a; Paidas et al., 2022b; Paidas et al., 2021; Jayakumar et al., 2014). After blocking with 5% BSA, membranes were incubated with specific antibodies. Primary antibodies targeting mitochondrial membrane proteins, including cyclooxygenase 1 (COX1; Cat# A23123, ABclonal, Woburn, MA, USA), mitochondrial translocase outer membrane (TOM-20; Cat# A19403, ABclonal, Woburn, MA, USA), and voltage-dependent anion channel (VDAC1; Cat# A19707, ABclonal, Woburn, MA, USA), were used at a 1:1,000 dilution and incubated at 4 °C. Anti-rabbit and anti-mouse horseradish-peroxidase-conjugated secondary antibodies (Vector Laboratories, Burlingame, CA, USA) were used at a 1:5,000 dilution. The optical density of the bands was measured using the Chemi-Imager (Alpha Inno-tech, San Leandro, CA, USA) digital imaging system, and the results were quantified with Sigma Scan Pro software as a ratio to the housekeeping protein β-actin.

#### RNA extraction

Total RNA was extracted from trophoblast cells using the miRNeasy Micro Kit (Cat #217084, Qiagen, Germantown, MD, USA) and quantified with a NanoDrop OneC spectrophotometer (Thermo Fisher Scientific, Waltham, MA, USA). RNA integrity was assessed using the Qubit High Sensitivity RNA Integrity and Quality (IQ) Assay Kit (Cat #Q33221, Thermo Fisher Scientific, Waltham, MA, USA).

#### mRNA sequencing and bioinformatics analysis

mRNA libraries were prepared using the NEBNext Ultra II directional RNA library prep kit (New England Biolabs Inc., Ipswich, MA, USA). Libraries were pooled and sequenced on the Illumina NovaseqX Plus platform with paired-end reads. Bioinformatic analyses were performed using DESeq2 (version 1.38.0). Differential expression of gene was assessed using DESeq2 (version 1.38.0), with a significance threshold of P < 0.05 and a log fold change of at least 1 (i.e., | log2 FC| ≥ 1), corresponding to twofold differences in expression between groups. Transcripts were considered differentially expressed when the adjusted p-values were below 0.05, fold changes exceeded ±1.5, and the false discovery rate (FDR)/adjusted p value was below 0.05. Gene Ontology (GO) and Kyoto Encyclopedia of Genes and Genomes (KEGG) analyses were conducted using the clusterProfiler package in R (version 4.4.0).

#### Real-time PCR

RNA (1 μg) extracted from trophoblast cells was reverse-transcribed into cDNA using a kit (Cat #11904–018, Invitrogen, Waltham, MA, USA) according to the manufacturer’s instructions. Genes were randomly selected, and primers were designed using Primer Bank, as listed in Table 1. Primers were obtained from Oligos (Sigma-Aldrich, Burlington, MA, USA). Quantitative real-time PCR was performed on an iCycler (Bio-Rad Laboratories, Hercules, CA, USA), and cycle thresholds were calculated using iCycler software version 3. Each sample was run in duplicate, and threshold values were normalized to the average CT of β-Actin.

**TABLE 1:** Primers for RT-PCR analysis.

Gene	Description	Forward primer sequence	Reverse primer sequence
Mfge8	Milk fat Globule-EGF factor 8	TCT​GTG​CGT​GTG​ACC​TTC​TTG	CCA​GGG​GTT​ATC​GTC​ATT​GCT
Oxct1	3-Oxoacid CoA-transferase 1	TGG​AGA​TGA​CGT​AAG​GGA​ACG	GGA​GAG​GGA​TTC​CTA​TGC​CCA
Grem1	Gremlin 1	CGG​AGC​GCA​AAT​ACC​TGA​AG	GGT​TGA​TGA​TGG​TGC​GAC​TGT
Cpa4	Carboxypeptidase A4	AGG​TGG​ATA​CTG​TTC​ATT​GGG​G	TTG​CTG​ATC​TCG​TCT​CCA​TTT​C
Ldha	Lactate dehydrogenase A	TTG​ACC​TAC​GTG​GCT​TGG​AAG	GGT​AAC​GGA​ATC​GGG​CTG​AAT
Pltp	Phospholipid transfer protein	CTC​TCC​ACG​TTC​ATC​ACC​TCA	AAT​GCC​AAC​AAG​CTC​GTC​CA
Col3a1	Collagen III	TTG​AAG​GAG​GAT​GTT​CCC​ATC​T	ACA​GAC​ACA​TAT​TTG​GCA​TGG​TT
Dram1	DNA damage regulated autophagy modulator 1	AGT​GCT​TGG​ATT​GGT​GGG​ATG	GAT​GGA​CTG​TAG​GAG​CGT​GTA
Mrc2	Mannose receptor C-type 2	CCG​AAA​CCG​GCT​ATT​CAA​CCT	CGG​TCA​CAC​TCA​TAC​ATG​CCC
Tfpi	Tissue factor pathway inhibitor	CTT​GCG​ACG​ATG​CTT​GCT​G	ACA​CCC​ACC​GGA​AAA​GAA​TTT
Atp2b1	Plasma membrane calcium-transporting ATPase 1	TGA​AGG​AGC​TGC​AAT​CCT​CTT	TGA​CCA​CCC​CTG​ATG​ACA​GT
Cfh	Complement factor H	CAC​ACA​AGA​TGG​ATG​GTC​GC	GGA​TGG​CAG​GCA​ACG​TCT​AT
Actb	βeta-Actin	GTC​GCG​GTC​ATC​AGG​AAC​TT	GTT​GAG​AAC​CGT​GTA​CCA​TGT

### Statistical analysis

Each experimental group included 4 to 6 wells (6-well plate) per condition for each assay. Western blot analyses used samples from 2 to 4 separate plates. All experiments were repeated with trophoblast cultures from 4 to 7 independent seedings. Fluorescence intensity and Western blot band intensities were quantified by densitometry. All the data were analyzed using GraphPad Prism version 9.5.1. Nonparametric comparisons were performed with Mann–Whitney U tests for cell Viability, immunofluorescence, Western blotting, and RT-PCR. Results with p-values less than 0.05 were considered statistically significant (*p < 0.05; **p < 0.01; ns p > 0.05).

## Results

### Mechanical trauma reduced cell viability in trophoblast cells

**FIGURE 1:**
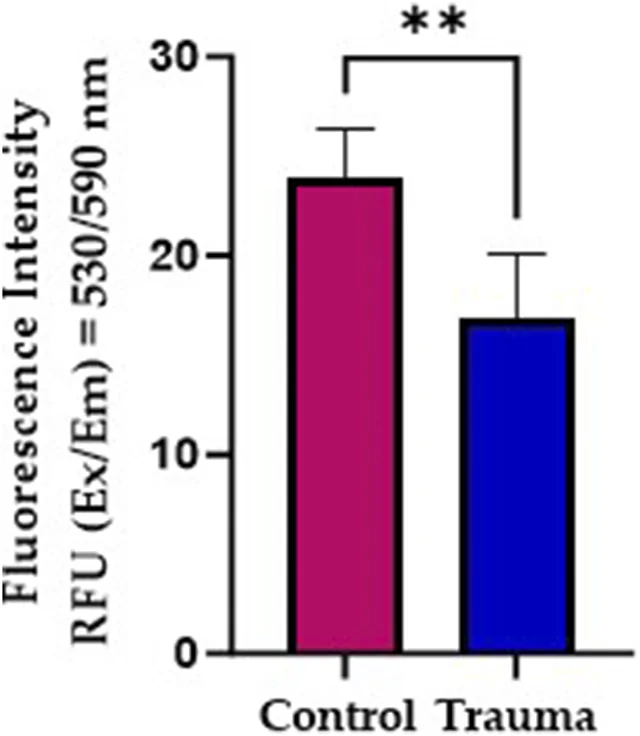
Trophoblast cell viability after trauma. Plot of the normalized fluorescence intensity as a function of cell viability. **indicates the statistical significance between control and trauma (p < 0.01). Data are expressed as the mean ± SEM (n = 6).

Cell viability, measured by fluorescence intensity, shows a significant reduction in traumatized cells compared to the control after normalization with protein (Figure 1). Fluorescence data confirm a notable decrease in cell viability, suggesting either impaired mitochondrial function or reduced cell viability.

### Mechanical trauma increases SDHA and sFlt-1 expression in trophoblast cells

**FIGURE 2:**
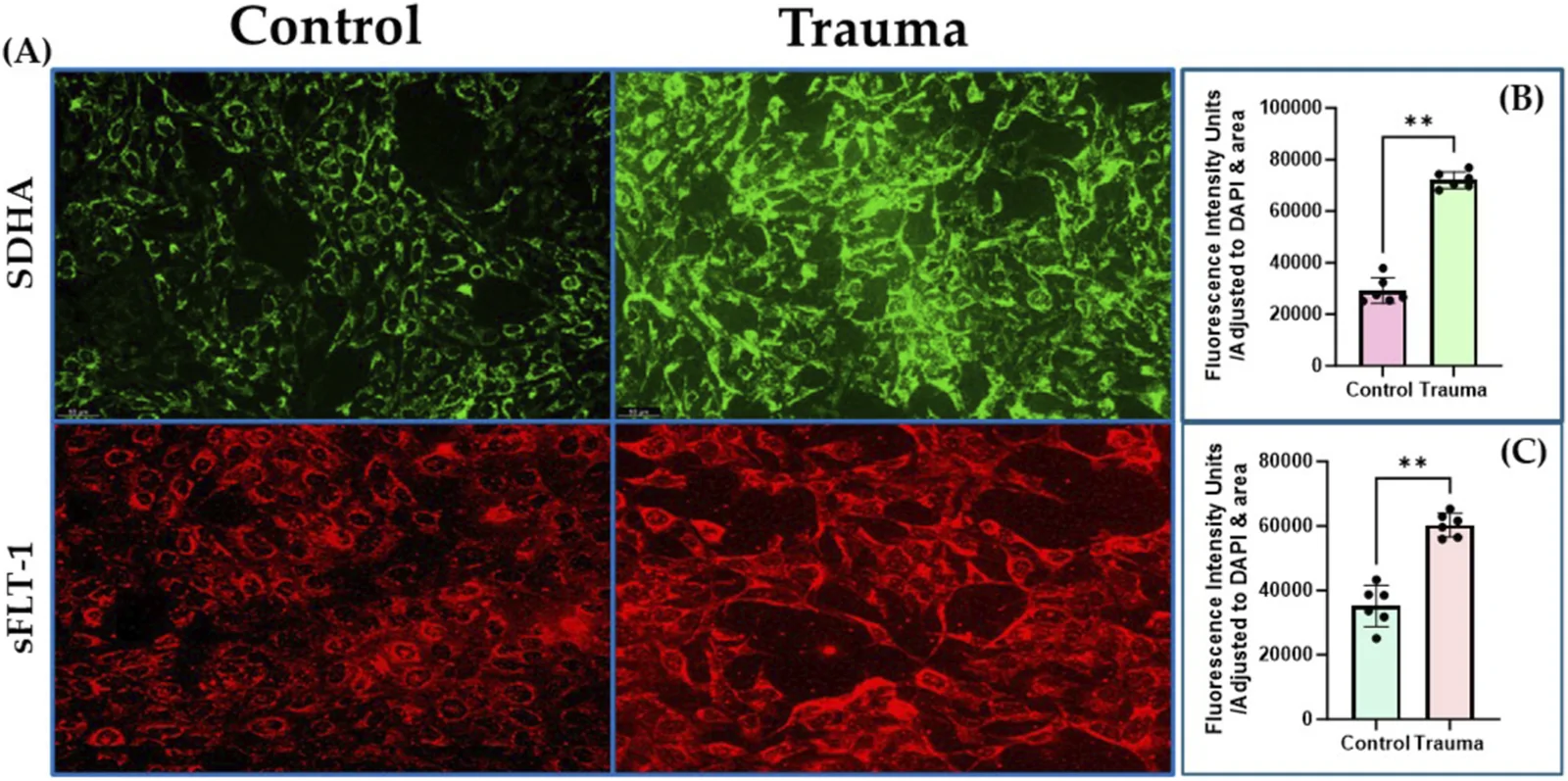
Alteration in the immunofluorescence levels of sFLT-1 and SDHA in the trophoblast cells post-trauma. **(A-C)** sFLT-1 (red) and SDHA (green) levels are significantly higher in the trauma group than in the control group. Values are the mean of six independent experiments. Scale bar = 50 μm **p < 0.01 is a statistically significant increase compared to the control (n = 6).

To further assess mitochondrial integrity, we analyzed the expression and co-localization of succinate dehydrogenase complex A (SDHA), a key component of the electron transport chain (Hu et al., 2023). We found increased SDHA expression in trophoblast cells that experienced trauma compared with those that did not (Figure 2A, green and Figure 2B, quantitation). Similarly, soluble fms-like tyrosine kinase-1 (sFlt-1), an antiangiogenic factor, was noticeably higher in the trauma group (Figure 2A red and Figure 2C quantitation). As a known marker of cellular stress and injury response, the rise in sFlt-1 indicates that trophoblasts become reactive after mechanical trauma (Charnock-Jones et al., 2004).

### Mechanical trauma reduces mitochondrial proteins in trophoblast cells

**FIGURE 3:**
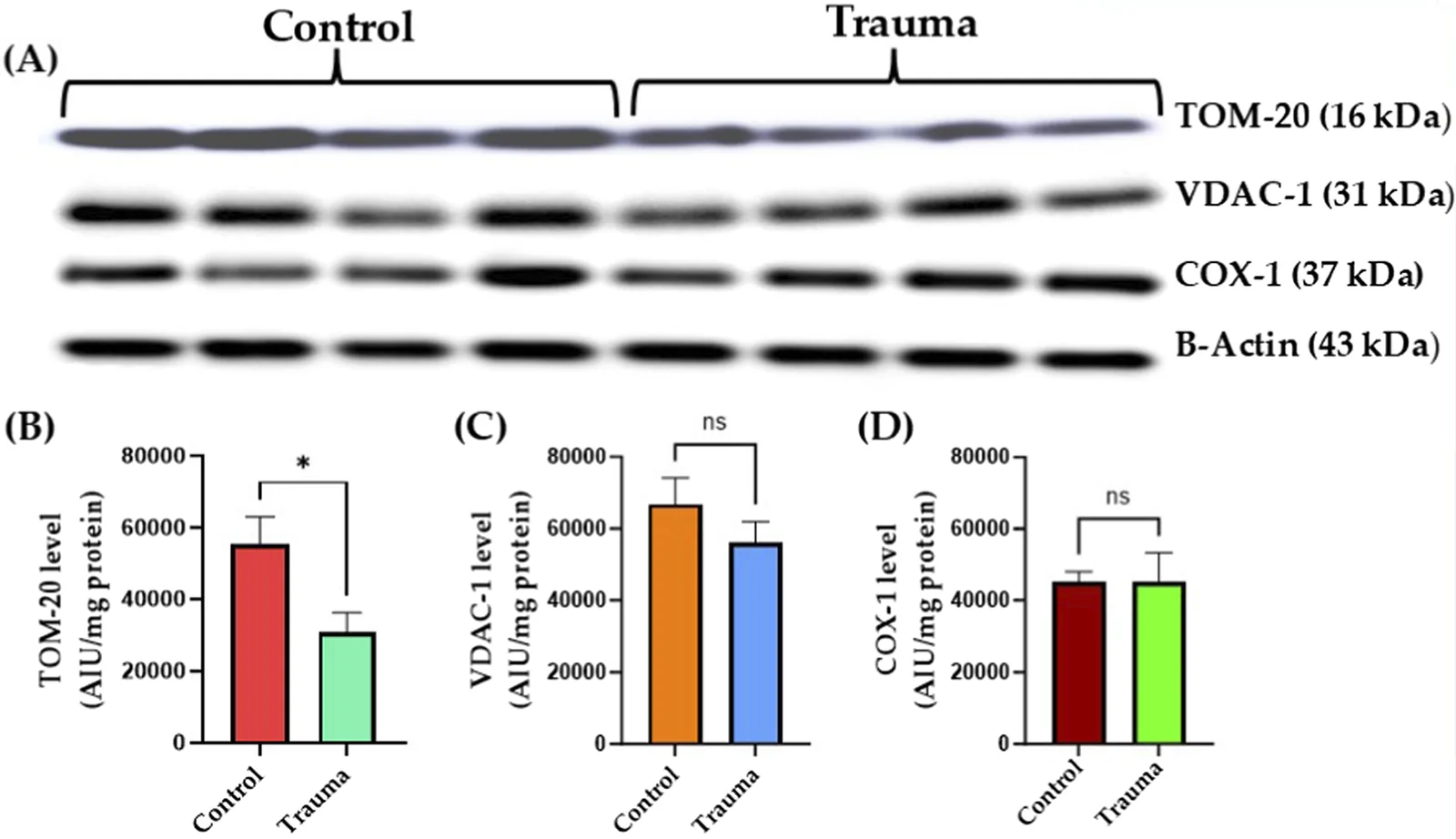
**(A)** Representative immunoblots show changes in TOM-20, VDAC-1, and COX-1 protein levels. **(B)** TOM-20 protein levels were significantly reduced in the trauma-induced trophoblast cells (p < 0.028). **(C)** VDAC-1 levels were not significantly reduced (p < 0.114), while. **(D)** COX-1 showed no change due to trauma (p < 0.685). Values are the mean with standard deviation (SD) of four independent experiments. * = p < 0.05 is statistically significant; ns = nonsignificant (n = 4).

Western blot revealed distinct changes in the expression of key mitochondrial membrane proteins in the traumatized trophoblast cells compared to normal/control cells (Figure 3A). We observed a slight reduction in VDAC-1 protein and a twofold decrease in TOM-20 protein levels in injured trophoblast cells compared to control (Figures 3B,C). Conversely, Cox-1 expression showed no change in response to trauma (Figure 3D).

### Mechanical trauma altered gene expression in trophoblast cells

**FIGURE 4:**
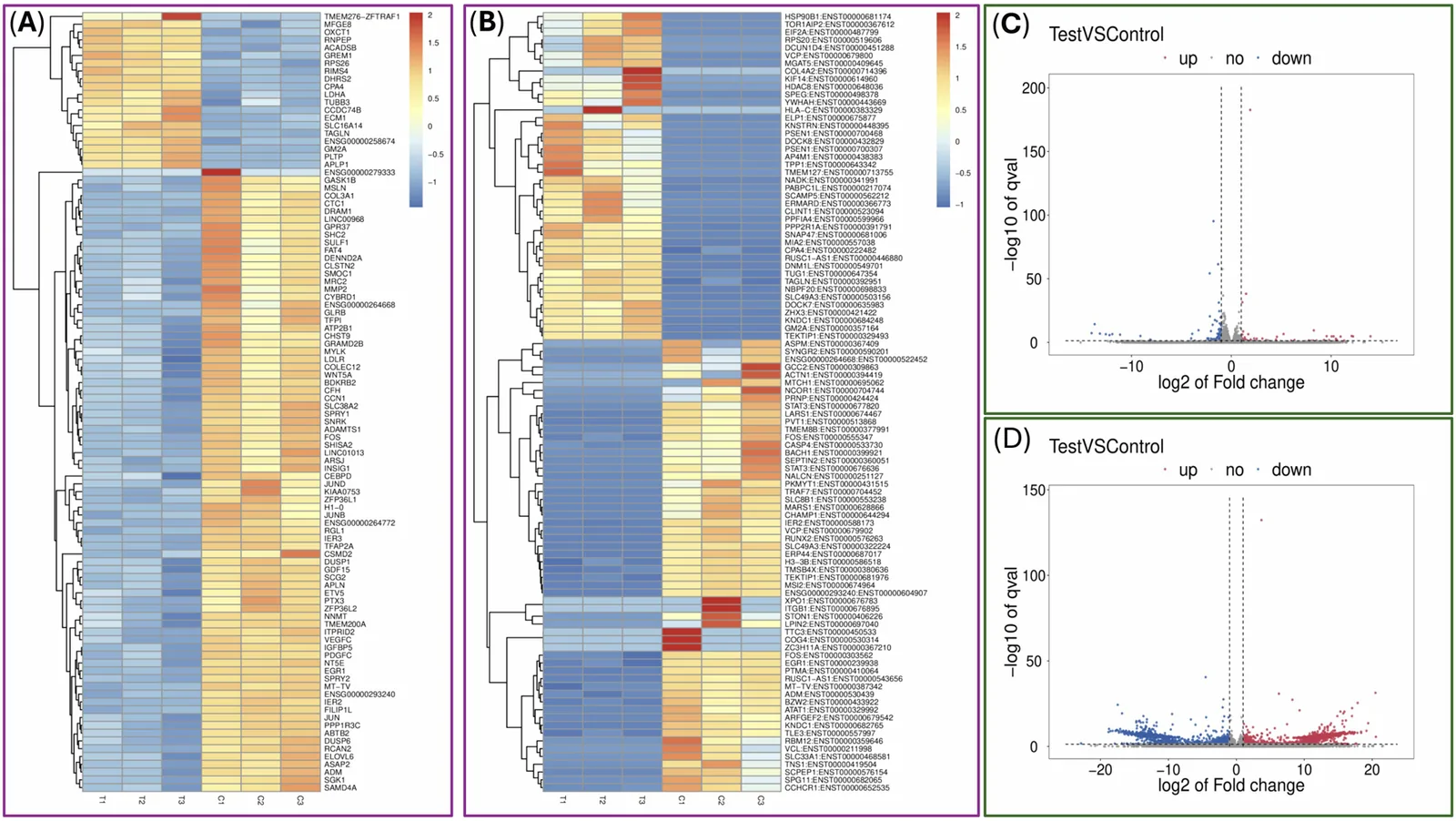
RNA-sequencing analysis reveals differential genes and transcript expression in traumatized trophoblast cells **(A)** Heatmap of DEGs across trauma (n = 3; T1–T3) and control (n = 3; C1–C3) samples. **(B)** Heatmap of DETs showing clustering patterns between trauma and control samples. **(C)** Volcano plot showing differentially expressed genes (DEGs) between trauma-treated and control trophoblast samples, log2 fold change vs. means of normalized counts (−log10; p-value) for DEGs (**p ≤ 0.05). **(D)** Volcano plot of differentially expressed transcript isoforms (DETs) between trauma-treated and control trophoblast samples, log2 fold change vs. means of normalized counts (−log10; p-value) for DETs (**p ≤ 0.05).

Differential expression analysis revealed many genes that were significantly changed between groups. Both upregulated and downregulated genes exhibited large fold changes, indicating extensive transcriptional reorganization after mechanical trauma (Figure 4).

Hierarchical clustering of differentially expressed genes clearly distinguished trauma-exposed from control trophoblast samples (Figure 4A). Several genes showed higher expression in traumatized trophoblast cells, including LDHA, MFGE8, ECM1, and TAGLN. Conversely, many genes showed lower expression than controls, including NNMT, MYLK, FOS, JUNB, CEBPD, EGR1, APLN, MMP2, COL3A1, VEGFC, CCN1, CFH, ATP2B1, DUSP1, SPRY1, and SPRY2. These differentially expressed genes are associated with multiple enriched functional categories and signaling pathways identified through GO and KEGG analyses. Transcript-level analysis showed similar differential patterns among transcript isoforms (4 B).

Volcano plot analysis revealed distinct expression patterns that separated trauma and control samples (Figures 4C,D). These findings indicate that mechanical trauma influences not only overall gene expression levels but also specific transcript isoforms, suggesting potential regulation via alternative splicing or transcript usage.

**FIGURE 5:**
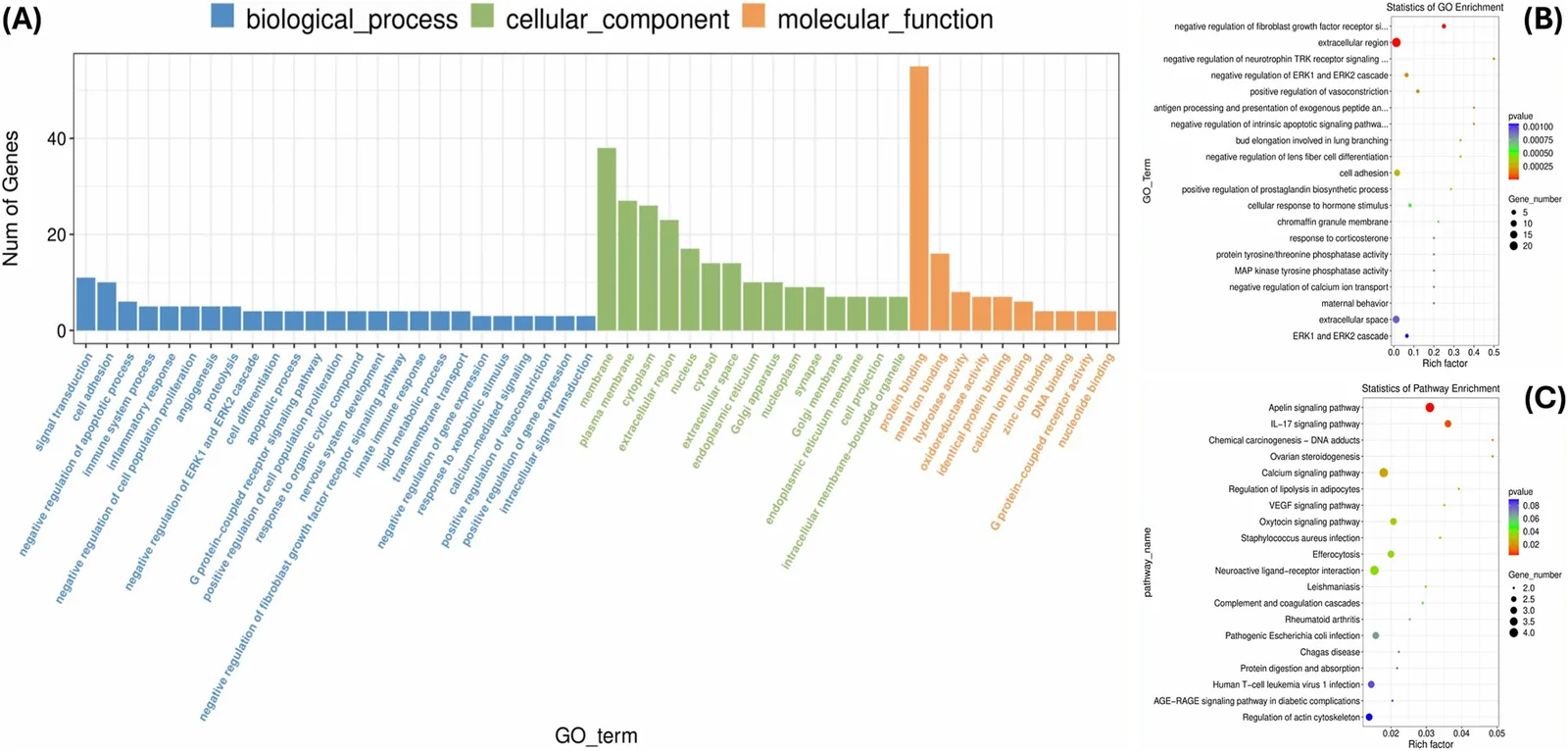
Functional enrichment analysis of differentially expressed genes following mechanical trauma in trophoblast cells. **(A)** GO classification of differentially expressed genes across Biological Process, Cellular Component, and Molecular Function categories. **(B)** GO enrichment analysis identifies significantly enriched functional terms **(C)** KEGG pathway enrichment analysis highlighting significantly enriched pathways.

Functional classification of differentially expressed genes using GO analysis revealed enrichment in biological processes such as signal transduction, cell adhesion, inflammatory response, angiogenesis, and apoptotic signaling. Enriched cellular component categories included membrane, plasma membrane, cytoplasm, extracellular region, and nucleus, indicating that many of these genes encode proteins involved in cellular signaling and extracellular interactions. Molecular function enrichment highlighted protein binding, metal ion binding, oxidoreductase activity, and hydrolase activity as the main functional classes (Figure 5A).

**FIGURE 6:**
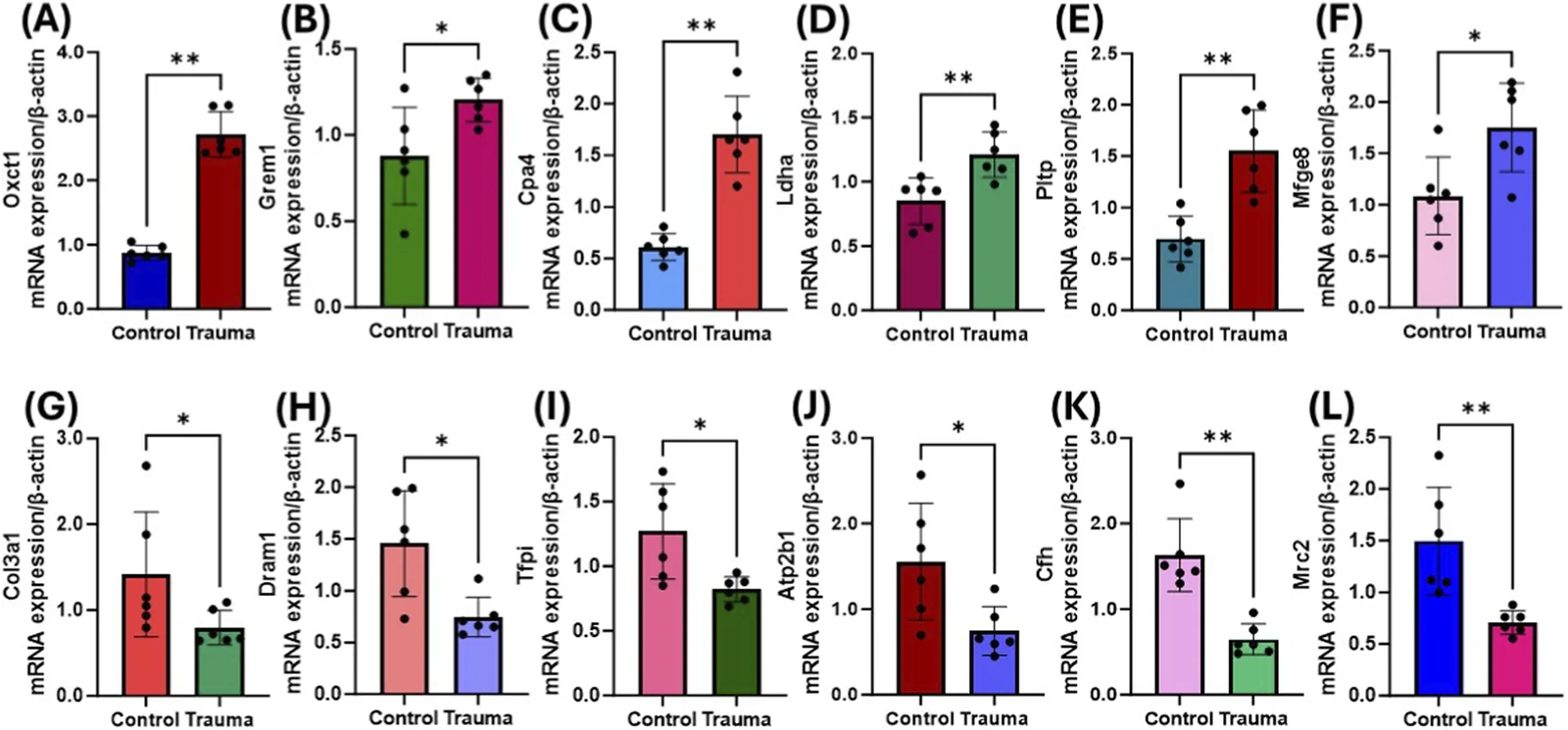
Significantly deregulated mRNA expression in the trophoblast cells post-trauma. **(A–F)** Significantly upregulated mRNA expression in traumatized trophoblast cells compared to control: **(A)** OXCT1; **(B)** GREM1; **(C)** CPA4; **(D)** LDHA; **(E)** PLTP; and **(F)** MFGE8 **(G–L)** Significantly downregulated mRNA expression in traumatized trophoblast cells compared to control: **(G)** COL3A1; **(H)** DRAM1; **(I)** TFPI; **(J)** ATP2B1; **(K)** CFH; and (L) MRC2. * = p < 0.05 is statistically significant; ** = p < 0.01 is statistically significant (n = 6).

GO enrichment analysis further identified several highly enriched functional terms (Figure 5B). Prominent enriched GO terms included regulation of the ERK1 and ERK2 cascade, cell adhesion, extracellular region, and cellular response to hormone stimulus. KEGG pathway enrichment analysis revealed several signaling pathways significantly affected by trauma, including regulation of the actin cytoskeleton, calcium signaling pathway, VEGF signaling pathway, complement and coagulation cascades, IL-17 signaling pathway, and apelin signaling pathway (Figure 5C). Differential gene expressions were further validated using RT-PCR. The results demonstrated a significant increase in the expression of OXCT1, GREM1, CPA4, LDHA, PLTP, and MFGE8 (Figures 6A–F), and a decrease in the expression of COL3A1, DRAM1, TFPI, ATP2B1, CFH, and MRC2 (Figures 6G–L).

## Discussion

This study provides the first comprehensive *in vitro* characterization of fluid percussion-mediated trauma using human trophoblast cells as a model to investigate trauma-induced pregnancy complications. Trophoblast trauma is associated with reduced cell viability, mitochondrial dysfunction, and disruption of angiogenic signaling. Additionally, RNA sequencing demonstrated coordinated alterations in genes involved in stress signaling, extracellular matrix organization, angiogenesis, inflammatory regulation, calcium homeostasis, and cellular metabolism, indicating that mechanical trauma disrupts multiple interconnected pathways required for normal trophoblast function.

### Trauma-induced mitochondrial dysfunction and anti-angiogenic signaling in trophoblast cells in vitro

Our findings show that mechanical trauma to cultured trophoblast cells results in significant mitochondrial dysfunction and reduced cell viability (Barak et al., 2023; Hussein et al., 2022; Ren et al., 2024). Specifically, we observed a decline in cell viability and a decrease in fluorescence intensity in traumatized trophoblasts, indicating impaired mitochondrial function and reduced metabolic activity. There was a notable rise in sFlt-1, an anti-angiogenic factor linked to placental problems. This increase in sFlt-1 after trauma supports the idea that trophoblasts activate a stress response following injury, which could affect angiogenic balance and placental function. Additionally, we found higher SDHA expression in traumatized trophoblast cells compared to controls. SDHA is involved in electron transport, helping to shuttle molecules to meet the high-energy demands of the cells. Increased SDHA expression was associated with shorter cell lifespan and increased cell death (Chattopadhyay et al., 2019).

Western blot analyses further revealed a distinct trauma-induced dysregulation of mitochondrial membrane proteins. We observed a moderate reduction in VDAC-1 and a more significant decrease in TOM20 protein levels in traumatized trophoblasts compared to controls. The decline in VDAC-1, a voltage-dependent anion channel located in the outer mitochondrial membrane, is vital for ATP production and regulation of apoptosis. VDAC-1 also functions as a scaffold for mitochondrial-mediated cell death, and its downregulation may heighten trophoblast vulnerability to oxidative or inflammatory stress (Chang et al., 2023; Lu and Sferruzzi-Perri, 2021).

TOM20, a key translocase of the outer membrane, is vital for importing nuclear-encoded proteins into mitochondria and is often used as a marker of mitochondrial content. The trauma-induced decrease in TOM20 aligns with mitochondrial loss or damage and reflects patterns seen in trophoblasts exposed to hypoxia-reoxygenation (Dhar-Mascareño et al., 2005) and in placentas from early-onset preeclampsia (Burton and Yung, 2011). Although earlier studies have documented compensatory increases in TOM20 during mitochondrial stress or turnover, the consistent reduction observed here, along with VDAC-1 loss, supports a model of overall mitochondrial depletion or dysfunction (Shah et al., 2015).

Increased sFlt-1 expression in traumatized trophoblasts further indicates disruption of endothelial signaling. sFlt-1 binds to VEGF and PIGF, preventing their interaction with endothelial receptors and hindering angiogenesis. Higher maternal circulating levels of sFlt-1 serve as a marker for preeclampsia and predict placental insufficiency and fetal growth restriction. Therefore, the rise in sFlt-1 seen *in vitro* may reflect the pathophysiological changes that occur *in vivo* after maternal trauma (Levine et al., 2004).

Decreased TOM20 and VDAC-1, elevated sFlt-1, and reduced viability indicate that mechanical trauma triggers a mitochondrial stress response in trophoblasts. These mitochondrial alterations are likely mediated by upstream signaling pathways, such as DRP1 hyperactivation, which has previously been shown to be driven by oxidative stress and to be associated with increased mitochondrial fission and fragmentation in trophoblasts under hypoxic conditions (Voros et al., 2025). Trauma-induced activation of DRP1 (via Ser616 phosphorylation) has been reported to impair mitochondrial bioenergetics and promote apoptosis in other models of placental injury (Gillmore et al., 2022).

Interestingly, the stable COX-1 expression suggests that not all mitochondrial regions are equally affected by trauma, possibly indicating selective vulnerability of the outer mitochondrial membrane compared to inner membrane components. This finding also supports the idea that mitochondrial dysfunction after trauma may occur before or independently of overall mitochondrial respiratory chain failure (Gilmer et al., 2009).

### Trauma-related differences in gene expression within trophoblast cells

To further characterize the molecular mechanisms underlying these protein-level changes, RNA sequencing was performed to determine whether mechanical trauma induced broader transcriptional alterations in pathways governing trophoblast function. The transcriptomic findings support and extend the protein analyses by demonstrating coordinated dysregulation of stress signaling, trophoblast remodeling, and cellular homeostasis.

#### The trophoblast fails to mount an effective adaptive response

Mechanical trauma not only altered mitochondrial integrity but also appeared to impair the trophoblast’s ability to mount an effective adaptive response to injury. GO enrichment analysis identified significant changes in the regulation of the ERK1/ERK2 cascade, a pathway that coordinates cellular responses to environmental stress, proliferation, differentiation, and survival. Rather than demonstrating activation of these protective transcriptional programs, traumatized trophoblasts exhibited coordinated downregulation of several immediate-early response genes, including FOS, JUN, CEBPD, and EGR1. Collectively, these transcription factors regulate trophoblast differentiation, migration, invasion, and adaptation to stress, and reduced expression has been associated with placental dysfunction and pregnancy-related disorders such as preeclampsia and recurrent spontaneous abortion (Renaud et al., 2014; Kubota et al., 2015; Bamberger et al., 2004; Chen et al., 2021; Hua et al., 2024).

Interestingly, disruption of this response extended beyond transcriptional activators. DUSP1, SPRY1, and SPRY2, which normally fine-tune MAPK signaling through negative feedback regulation, were also downregulated following trauma (Xu et al., 2021; Andrieu et al., 2025; Hanafusa et al., 2002). Although these genes typically function as inhibitors of ERK signaling, their concurrent reduction alongside ERK-responsive transcription factors suggests that trauma does not simply activate or inhibit a single signaling pathway. Instead, these coordinated changes point toward a broader collapse of the regulatory network responsible for sensing cellular stress and orchestrating trophoblast adaptation. This interpretation is consistent with our protein-level findings of mitochondrial dysfunction, reduced viability, and increased markers of cellular stress, suggesting that mechanical trauma compromises both the structural integrity of trophoblasts and their capacity to initiate protective transcriptional responses following injury.

#### The trophoblast loses its functional phenotype

Beyond impairing adaptive stress signaling, mechanical trauma also appeared to compromise the specialized functions required for normal trophoblast behavior. Successful placentation depends on coordinated regulation of trophoblast migration, extracellular matrix remodeling, and communication with the developing placental vasculature. Transcriptomic analysis demonstrated widespread suppression of genes supporting these processes, suggesting that injured trophoblasts progressively lose the molecular characteristics required to maintain normal placental development.

Several genes critical for trophoblast invasion and extracellular matrix remodeling, including MYLK, MMP2, and COL3A1, were significantly downregulated following trauma. MYLK regulates actomyosin contractility during trophoblast migration, MMP2 facilitates extracellular matrix degradation necessary for invasion, and COL3A1 contributes to structural organization of the placental villous stroma (Zhao et al., 2022; Lee et al., 2019; Arai and Nishiyama, 2007). The reduced expression of these genes suggests impaired matrix remodeling and trophoblast motility after trauma. Loss of normal cell-matrix interactions can trigger anoikis, a form of detachment-induced apoptosis, potentially contributing to decreased trophoblast viability (Mei et al., 2020). Simultaneously, expression of multiple angiogenic regulators, including VEGFC, CCN1, and APLN, was reduced, suggesting impaired trophoblast communication with the developing placental vasculature (Dunk and Ahmed, 2001; Mo et al., 2002; Van Mieghem et al., 2016). Together, these coordinated transcriptional changes suggest that trauma not only limits trophoblast motility but may also impair the molecular signaling required to support placental vascular development.

Not all structural genes followed this pattern, however. TAGLN, MFGE8, and ECM1 were upregulated after trauma, despite the broader suppression of other invasion- and angiogenesis-associated genes. Rather than indicating preserved trophoblast function, increased expression of these genes may represent compensatory attempts to stabilize cytoskeletal organization and cell-matrix interactions following injury (Liang et al., 2019; Wang et al., 2022; Muhandiram et al., 2024). Similarly, ECM1 has been identified among the most highly expressed proteins in recurrent pregnancy loss and has been associated with angiogenesis, cell migration, and blood circulation pathways (Tóth et al., 2025). The increased expression of these genes may represent an attempt by injured trophoblasts to stabilize cytoskeletal organization and maintain cell-matrix interactions following mechanical injury. However, these compensatory responses appear insufficient to overcome the broader suppression of pathways required for normal trophoblast invasion and placental remodeling, suggesting that trophoblasts enter a state of structural compensation but functional impairment following mechanical trauma.

#### Mechanical trauma disrupts trophoblast homeostasis

Following the loss of adaptive stress signaling and impairment of trophoblast remodeling, our transcriptomic findings suggest that mechanical trauma further disrupts the cellular processes required to maintain trophoblast homeostasis. Enrichment of the complement and coagulation cascade, calcium signaling, IL-17 signaling, and metabolic pathways indicates that trauma affects multiple interconnected regulatory systems responsible for preserving immune balance, intracellular signaling, and cellular metabolism rather than isolated biological processes.

One consequence of this disruption was altered regulation of immune homeostasis. CFH, a major inhibitor of complement activation, was significantly downregulated following trauma and this reduction was confirmed by RT-PCR analysis. Under normal conditions, CFH protects placental tissues from complement-mediated injury by preventing excessive activation of the alternative complement pathway (Yasmin et al., 2024). Reduced maternal CFH levels have been associated with spontaneous preterm birth, supporting the importance of complement regulation during pregnancy (Becerra-Mojica et al., 2024). In addition, the downregulation of DUSP1, which has previously been linked to inflammatory MAPK signaling and macrophage polarization, further suggests that trauma may shift trophoblasts toward a dysregulated inflammatory environment capable of impairing normal placental function (Wu et al., 2024).

Disruption of cellular homeostasis was also evident in pathways regulating intracellular calcium balance. ATP2B1, which encodes the plasma membrane calcium ATPase responsible for maintaining intracellular calcium concentrations, was downregulated in both RNA sequencing and RT-PCR analyses. Calcium signaling is fundamental for trophoblast migration, differentiation, mitochondrial function, and cell survival (Marín et al., 2008). Similar disturbances in calcium homeostasis have been reported in preeclampsia and intrauterine growth restriction (Baczyk et al., 2011), while altered calcium signaling has been linked to activation of apoptotic pathways within trophoblasts (Bolnick et al., 2014). These findings suggest that trauma-induced disruption of calcium regulation may further compromise trophoblast viability by impairing intracellular signaling pathways essential for maintaining normal cellular function.

Finally, trauma was accompanied by evidence of metabolic reprogramming. LDHA was consistently upregulated in both RNA sequencing and RT-PCR analyses, suggesting increased reliance on glycolytic metabolism following injury. Similar increases in LDHA have been described in trophoblasts exposed to hypoxic stress through HIF-1α activation (Kay et al., 2007), and dysregulated glycolysis has been implicated in pregnancy complications including miscarriage and preeclampsia (Gou and Zhang, 2023). In contrast, NNMT was significantly downregulated after trauma. Reduced NNMT expression has been shown to impair trophoblast proliferation, migration, and invasion through the COMP/CD36/ERK1/2 signaling axis and has been reported in recurrent spontaneous abortion as well as in trophoblasts exposed to hypoxic and inflammatory conditions (Huang et al., 2025; Tossetta et al., 2025). Together, these findings suggest that mechanical trauma is associated with metabolic adaptation to cellular stress while simultaneously impairing pathways required to sustain normal trophoblast function. When considered alongside the observed mitochondrial dysfunction and suppression of adaptive stress signaling, these metabolic changes further support a model in which mechanical trauma progressively compromises trophoblast homeostasis and survival.

**FIGURE 7:**
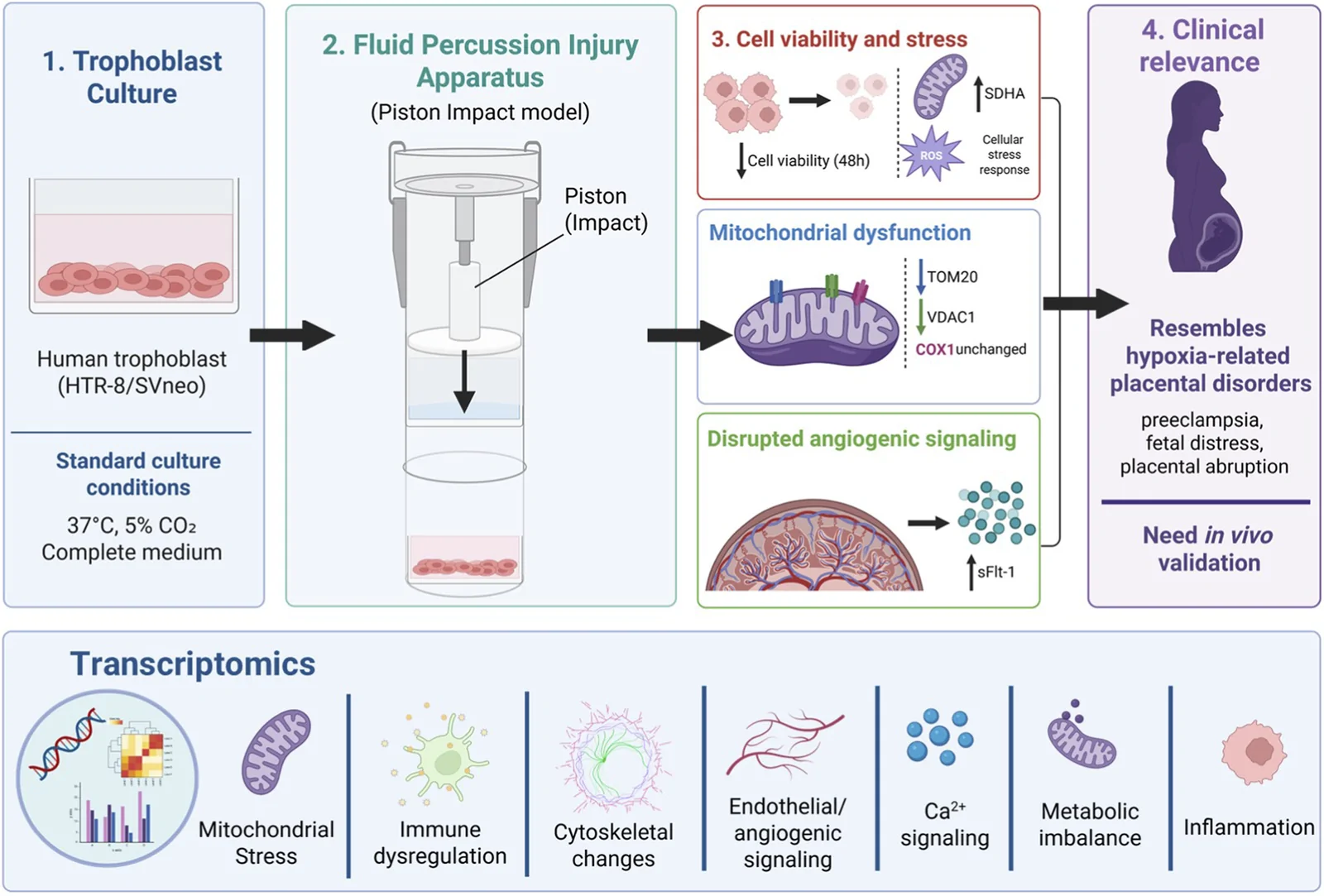
Schematic representation illustrates how trauma may lead to problems with trophoblast function.

Taken together, our findings support a model in which mechanical trauma to trophoblasts causes mitochondrial dysregulation characterized by reduced outer membrane protein expression (Tom20, VDAC1), increased anti-angiogenic signaling (sFlt-1), and impaired cell survival. The accompanying transcriptomic alterations suggest that these protein-level changes are part of a broader biological response in which trophoblasts exhibit impaired adaptive stress signaling, disrupted extracellular matrix remodeling, loss of angiogenic support, and progressive dysregulation of cellular homeostasis. Rather than representing isolated molecular abnormalities, these coordinated changes indicate that mechanical trauma affects multiple interconnected pathways required for normal trophoblast function (Figure 7). These molecular and cellular changes are similar to those seen in other models of placental injury and imply that trauma may initiate a pathological process contributing to adverse pregnancy outcomes such as preeclampsia, fetal growth restriction, and preterm birth. These findings provide new mechanistic insight into the molecular responses of trophoblasts following mechanical injury and establish a foundation for future studies investigating therapeutic strategies aimed at preserving placental function after maternal trauma. Future research should explore how long these changes last, whether they can be reversed, and if targeting mitochondrial health or sFlt-1 signaling could offer therapeutic benefits in pregnancies impacted by trauma.

Limitations: First, our assessment of mitochondrial alterations after trauma in trophoblast cells is limited to TOM20 and VDAC expression, as well as cell viability assays. Although these markers reflect mitochondrial membrane integrity, additional assessment of mitochondrial dynamics, bioenergetics, and oxidative stress would provide a more comprehensive understanding of trauma-induced mitochondrial dysfunction. Second, this study focused on cultured trophoblasts; it remains unclear how well these findings apply to the *in vivo* placenta after trauma. The placenta is a complex organ made up of various cell types, and trophoblasts are just one part of the maternal-fetal interface. Additionally, systemic inflammatory responses, hormonal signals, and hemodynamic changes caused by trauma might further influence placental mitochondrial function. Although several differentially expressed genes were validated by RT-PCR, most transcriptomic findings were not assessed at the protein level, and future studies should determine whether these transcriptional changes translate to corresponding functional protein alterations. In addition, these cellular responses were measured at 1 time point (48 h) after mechanical trauma; future research is needed to determine whether these initial changes in viability, mitochondrial function, and gene expression persist, improve, or worsen over time. Using a 2D model is also a significant limitation given the human placenta’s complex, three-dimensional, and heterogeneous structure. Future studies involving *in vivo* models, placenta explants, or 3D models across both acute and chronic time points could help confirm our results and better define the temporal progression of trauma-induced placental injury.

## Conclusion

This study shows that mechanical trauma causes significant mitochondrial dysfunction and cellular stress in human trophoblasts, as indicated by decreased viability, lower expression of TOM20 and VDAC1, and increased levels of the antiangiogenic factors sFlt-1 and SDHA. These results suggest that trauma activates a mitochondrial stress response similar to those seen in placental disorders such as preeclampsia. The loss of key mitochondrial outer membrane proteins may indicate early mitochondrial damage, while elevated sFlt-1 may signal downstream disruptions in angiogenic signaling, and increased SDHA may lead to cell death. Transcriptomic analysis further demonstrated that these protein-level alterations are accompanied by coordinated changes in pathways regulating adaptive stress responses, trophoblast remodeling, angiogenesis, immune regulation, calcium homeostasis, and cellular metabolism. Together, these findings support a model in which mechanical trauma progressively impairs the trophoblast’s ability to respond to injury, maintain normal placental functions, and preserve cellular homeostasis. This integrated molecular response provides a potential mechanistic explanation for how maternal trauma may contribute to placental dysfunction and adverse pregnancy outcomes. Future *in vivo* studies are needed to confirm these mechanisms and explore potential therapies to maintain placental mitochondrial health in pregnancies affected by trauma.

## Data Availability

The data from this study are available upon request from the corresponding authors.
